# A prospective study of a new prediction model of vaginal birth after cesarean section at a tertiary care centre

**DOI:** 10.4274/tjod.galenos.2020.82205

**Published:** 2020-12-10

**Authors:** Pinkey Lakra, Bhagyashri Patil, Sunita Siwach, Manisha Upadhyay, Shivani Shivani, Vijayata Sangwan, Rajiv Mahendru

**Affiliations:** 1 Bhagat Phool Singh Government Medical College for Women, Khanpurkalan, Sonepat, Haryana, India

**Keywords:** Vaginal birth after a cesarean, prediction, model

## Abstract

**Objective:**

To create a new and simple model for predicting the likelihood of vaginal birth after cesarean (VBAC) section using variables available at the time of admission.

**Materials and Methods:**

A prospective observational study was performed at a tertiary care centre in Haryana over a period of 12 months (January 2018 - December 2018) in pregnant women attending the labour room with one previous cesarean section fulfilling the criteria for undergoing trial of labour after cesarean (TOLAC). The sample size was 150. A VBAC score was calculated for each patient using a new prediction model that included variables available at the time of admission such as maternal age, gestational age, Bishop’s score, body mass index, indication for primary cesarean section, and clinically estimated fetal weight. The results of the VBAC scores were correlated with outcomes i.e. successful VBAC or failed VBAC. The chi-square test and Student’s t-test was used for comparison among the groups. Descriptive and regression analysis was performed for the study variables.

**Results:**

Out of 150 TOLAC cases, 78% had successful VBAC and the remainder (22%) had failed VBAC. The observed probability of having a successful VBAC for a VBAC score of 0-3 was 34%, 4-6 was 68%, 7-9 was 90%, and ≥10 was 97%. The prediction model performed well with an area under the curve of 0.77 (95% CI: 0.68 to 0.85) of the receiver operating characteristics receiver operating characteristic curve.

**Conclusion:**

The present study shows that the proposed VBAC prediction model is a good tool to predict the outcome of TOLAC and can be used to counsel women regarding the mode of delivery in the current and subsequent pregnancies. Further studies of this model and other such models with different permutations and combinations of variables are required.

**PRECIS:** A new prediction model of vaginal birth after cesarean containing factors available at the time of admission was tested and it was found to be a good tool.

## Introduction

The effective and safe use of cesarean delivery has been a focus and concern for last the three decades. It was the result of the 1980 National Institutes of Health Consensus Conference on Cesarean Childbirth held in response to the three-fold increase in the rate of cesarean deliveries (from 5% in 1970 to 15.2% in 1978) that vaginal birth after cesarean (VBAC) came into being^(1)^. As a result, the VBAC rate rose from 19.9% in 1990 to 28.3% in about a decade and the cesarean delivery rate decreased from 22.7% to 20.7%^(2)^. Later, with increasing incidence of uterine rupture, VBAC had a setback and went into disrepute, once again leading to an increase in the cesarean rates. This has led to significant research in determining the best permutations and combinations of the factors to achieve the optimum outcome of a previous cesarean delivery. There are numerous factors such as maternal age, body mass index (BMI), gestational age, spontaneous or induced labor, inter-conception period, estimated fetal weight, Bishop’s score, type of previous cesarean scar, and indication for primary cesarean delivery, which can influence the decision to undergo a trial of labour after cesarean (TOLAC) and its outcome i.e. failed VBAC (emergency repeat cesarean section) or successful VBAC (vaginal delivery).

Rates of maternal complications are highest among women who attempt vaginal birth and fail, intermediate among women who have planned cesarean delivery, and lowest among women who attempt vaginal birth and succeed^(3)^. VBAC success rates also vary between institutions and service providers. Thus, it is worth remarking that as of now, there is no reliable and demonstrable algorithm or nomogram that correctly identifies or accurately predicts the success of VBAC^(4)^. Hence, management of a case of previous lower segment cesarean section continues to be an obstetric dilemma.

Therefore, an accurate and reliable prediction model must be designed and validated to predict a successful outcome, but literature is scarce from India that could assess the ante-partum and intrapartum determinants for predicting successful VBAC. Hence, this study was planned. The aim of the study was to create a new model for predicting the likelihood of VBAC using variables available at the time of admission.

The objective of the study was to test the performance of the prediction model for success of VBAC delivery.

## Materials and Methods

This prospective observational study was conducted at a tertiary care centre over a period of 12 months (January 2018 to December 2018) in pregnant women attending the labour room with one previous cesarean section. This hospital a referral center for three major districts of Haryana State in North India with annual live birth rates ranging from 4,500 to 5,000, average overall cesarean rate of 20-25% of total deliveries, and a repeat cesarean rate of 30-35% of total cesareans. At 5% alpha error, 80% power and 95% confidence interval (CI), the sample size calculated using Master 2.0 software (India) was 150. Ethical committee approval of the study was obtained (approval number: BPSGMCW/RC279/IEC/18) and informed and written consent was given by each patient who fulfilled the following inclusion and exclusion criteria to undergo TOLAC.

**Inclusion criteria: **Singleton pregnancy, vertex presentation, one prior LSCS with non-recurrent indication, gestational age ≥37 weeks confirmed in first-trimester scan and menstrual history, maternal age (18-35 years).

**Exclusion criteria: **Age <18 years or >35 years, intrauterine fetal death, lethal fetal anomalies, non-reassuring fetal heart rate on admission, cephalopelvic disproportion, malpresentation, history of antepartum haemorrhage or adherent placenta in the current pregnancy,  history of uterine surgery other than cesarean section.

The following system was designed using the relative weights of significant factors used in previous models given by Troyer and Parisi et al.^(5)^, Flamm and Geiger^(6)^, Grobman et al.^(7,8)^, Wen et al.^(9)^, and Metz et al.^(10)^. In the proposed model, we included six variables, four of which, namely maternal age in years, gestational age in weeks, indication for primary cesarean, and BMI were also included in Grobman’s model. In place of cervical dilation, we used Bishop’s score, which was the main factor in the study by Metz et al.^(10)^ Estimated fetal weight was the sixth variable, which was also used in the study by Wen et al.^(9,10)  ^There are only a few Indian studies on prediction models and most studied individual factors instead of prediction models, which is why statistically significant factors previously studied in Indian studies as well as supported by the American College of Obstetrics and Gynecology and the Royal College of Obstetrics Gynaecology guidelines were included^(3,11,12,13)^. Each variable used in the prediction model was assigned a score of 0, 1, or 2. Scores were decided based on the previous models e.g Flamm and Geiger^(6) ^and Troyer and Parisi et al.^(5)^ who gave a score of 0 to those with a primary indication of cesarean section as failure to progress^(12,14)^. A score of 2 was chosen for breech and fetal distress because, according to the literature, this group had a statistically significant favourable VBAC outcomes in TOLAC studies. Similarly, other factors were given scores accordingly. A pilot study of this model was performed on 50 patients, the results were analyzed, necessary corrections were made, and later it was performed on 150 more patients.

### VBAC scoring system used in the proposed prediction model:

1. Maternal age (in years): a. >30=0    b. 25-30=1c.18-25=2

2. Gestational age (in weeks): a. <39=0 b. 39-40=1 c. >40=2

3. Indication for primary caesarean-section:

a. Non-progress of labor (NPOL) and others = 0

b. Intrauterine growth restriction (IUGR), oligohydramnios, Antepartum haemorrhage =1

c. breech presentation or fetal distress =2

4. Bishop score: a. 0-3=0 b. 4-5=1 c. 6-10=2

5. BMI in kg/m^2^ on admission:

a. 30=0 b. 25-29=1 c. <25=2

6. Clinically estimated fetal weight in grams according to Johnson’s formula:

a. >3500=0  b. 2500-3500=1c. <2500=2

VBAC scores were calculated for each patient fulfilling the criteria to undergo TOLAC at the time of admission and the result obtained was correlated with the outcome i.e. failed VBAC or successful VBAC.

### Statistical Analysis

Statistical package for the social sciences version 20 was used for statistical analysis. Descriptive statistics were used for the demographic features such as age, parity, gestational age, BMI, Bishop’s score, and the indication for primary cesarean section. The chi-square test and Student’s t-test was used for comparisons among the groups. Multivariate logistic regression analysis using the enter method was performed to calculate the adjusted odds ratio for each factor used in the VBAC prediction model to determine their association with successful VBAC. A p-value of ≤0.05 was considered statistically significant. Finally, the receiver operating characteristic (ROC)  curve was measured by calculating the corresponding area under the curve (AUC) and 95% CI.

## Results

Out of 150 TOLAC cases, 78% had successful VBAC and the remainder (22%) had failed VBAC (emergency repeat cesarean section). Table 1 depicts the individual variables of the VBAC prediction model, their frequency distribution, and their means with standard deviation. Table 2 shows the indications of cesarean section in the failed VBAC group. The most common indication was fetal distress followed by scar tenderness and failed induction in 27% (9/33), 25% (8/33), and 21% (7/33), respectively. Out of eight cases of scar tenderness, three had thinned out scar of previous cesarean section.

**Graph 1:**
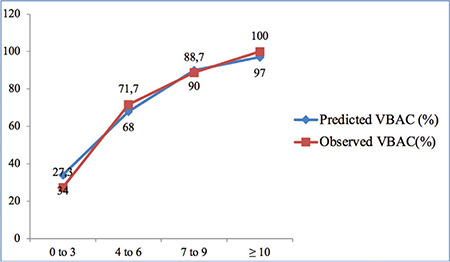
Predicted compared with observed vaginal birth after cesarean section (VBAC) (Successful TOLAC)

We developed a total score of 0-12. The final cumulative VBAC score ranged from 2 to 11 in the present study. It was 10 or more in 5.30%, 7 to 9 in 47.3%, 4 to 6 in 40.10%, and 3 or less in 7.30% of the cases. As can be seen in graph 1, the observed probability of having a successful VBAC for VBAC score 0-3 was 34%, 4-6 was 68%, 7-9 was 90%, and ≥10 was 97%. The predicted VBAC percentages also mentioned in graph 1 were calculated using binary logistic regression analysis and were closely related to the observed ones.

The multivariate regression analysis of all six variables as depicted in the Table 3 shows that two variables i.e. gestational age and Bishops score had the odds of 2.047 and 3.082, respectively, for having a successful VBAC with significant p-values in both, and with each 1 unit increase in BMI the odds of having a successful VBAC reduced by 0.832 (p<0.05). The other three factors i.e. age, indication for previous cesarean, and estimated fetal weight (Johnson’s formula) were not significantly associated with successful VBAC.

As shown in Table 4, we studied five additional variables out of the model variables, namely spontaneous onset of labour, parity, interdelivery interval in months, previous history of successful VBAC, and previous history of normal vaginal delivery. These variables were chosen because they were also included in the previous models by Grobman and Flamm, Geiger and Wen et al.^(6,8,9,10)^. Out of these five, two i.e. spontaneous onset of labour and parity were significantly associated with successful VBAC with odds of 2.58 and 5.138, respectively; the remaining three had no significant effect.

Seventeen (11.3%) patients out of 150 who underwent TOLAC had complications such as neonatal intensive care unit admissions (n=6), failed and successful VBAC groups (n=3 for both), and bladder injury (n=1) in the failed VBAC group, and a total of 10 cases of PPH (all atonic PPH), four in the successful VBAC group and six in the failed VBAC group. There were no cases of uterine rupture and ICU admissions in the present study.

**Figure 1:**
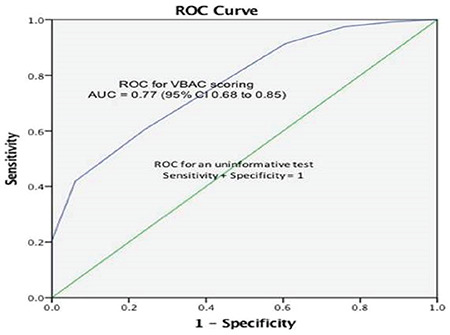
ROC curve for the scoring system showing area under the curve (AUC) ROC: Receiver operating characteristic curve

Figure 1 shows the ROC curve with an AUC equal to 0.77 (95% CI: 0.68 to 0.85).

## Discussion

The ACOG 2010 quoted the success rate of VBAC as 60-80%^(3)^. VBAC success rates also vary between institutions and service providers. The success rate of VBAC in the present study was 78%, similar to the quoted percentage. The mean age of the women in the present study was 25.84±4.20 years and there was no significant difference in the ages of the women in the two groups i.e. successful VBAC and failed VBAC groups, similar to study by Metz et al.^(10)^ (27.9±4.3 and 27.5±4.6; p=0.20). The mean age in the current study was lower as compared with the study by Xing et al.^ (15)^ because 76.6% of the women were from rural areas where young marriage and childbirth is common; the mean age in the successful VBAC and failed VBAC group was higher and there was a significant difference between the two groups (27.9±4.8 and 35.5±4.7; p=0.043).

Troyer and Parisi^(5)^ in 1992 studied 264 women with one previous cesarean section and developed a model with four factors, namely previous dysfunctional labour, non-reassuring fetal heart tracing at admission, no previous vaginal delivery, and labour induction. Each variable was given one point and the total score ranged from 0-4. The patients with the lowest score (i.e. 0) had the highest VBAC success rate (91.5%) as compared with those with higher scores. This model has not been studied extensively and needs further research and validation. The indication of primary cesarean section i.e. previous dysfunctional labour was also included in the present study, mentioned here as NPOL, and was similarly assigned a zero score.

There is a popular model known as the Flamm scoring system, which was developed in 1997 in California^(6)^. The research was performed on 5022 pregnant women including four variables known at the time of admission i.e. age of the patient, vaginal delivery before and after the cesarean section, a non-recurring indication of primary caesarean, cervical dilatation and cervical effacement. The result was given as a score of 0-10 and each score had a different percentage of success i.e. 0-2 corresponded to 49.1%, 3-7 corresponded to 59.9%, 66.7%, 77%, 88.65%, and 92.65%, and the success of 8-10 was 94.9%. In the present study, we used two of the above factors in the VBAC prediction model i.e. patient age, but instead of cervical dilation, we used Bishop’s score.

Patel et al.^(12)^ from Gujarat (India) in 2016 performed a prospective observational study on 150 women with one previous cesarean section using the Flamm scoring system. They found a mean Flamm score for successful VBAC of 5.35 (95% CI: 3.9 to 6.7) compared with failed VBAC i.e. 3.62 (95% CI: 27 to 4.57) and the chances of success of TOLAC increased with increasing Flamm scores. Similarly, in present study, the mean VBAC score of the failed VBAC group was 5.03±1.82 and that of the successful VBAC group was 7.01±1.77 (95% CI: 1.28 to 2.67; p<0.001). The authors concluded that Flamm scoring gave a fair judgment of successful vaginal birth in TOLAC, and using Flamm scores and monitoring though partogram would reduce the rate of cesarean sections in patients with one previous lower segment cesarean section. However, this model also has limited supprting data and needs further validation.

The most studied prediction model of VBAC was developed by Grobman of the northwestern University of Chicago in 2007^(8)^. It included six variables: maternal age, BMI, ethnicity (e.g. African-American/Hispanic), any previous vaginal delivery, any vaginal delivery since the last cesarean, and indication for primary cesarean of the arrest of dilation or descent. All variables were those that could be determined at the first antenatal visit with the idea of starting the counselling in the first trimester. Later, this model was improved in 2009 by adding certain other factors like most recent BMI within 2 weeks of delivery, gestational age at delivery, gestational diabetes mellitus, preeclampsia, cervical examination findings at admission, and the undertaking of labour induction. The result was expressed as the percentage of success of TOLAC. Inclusion of these additional factors slightly improved the performance of the calculator. Similarly, we tried to include those variables in the present prediction model, which were available at the time of admission because our hospital is a referral hospital and most of the time our patients are unscheduled (77.3% in the present study).

The AUC of the ROC curve of Grobman’s 2007 model was 0.751, and that of the new model was 0.779, and these values were significantly different (p<0.001)^(9)^. This model is currently known as the MFMU calculator and is freely available on the internet. The AUC of the ROC curve of our model was 0.77, similar to that of Grobman’s 2007 and 2009 models (0.751 and 0.779), which suggests that the proposed prediction model performed well.

In 2018, Wen et al.^(9)^ conducted a retrospective cohort study on 444 women with one cesarean delivery and at least one subsequent attempt for a trial of labor in Nanjing, China. They used Grobman’s model and also a modified version of this model and compared the two. The considered potential VBAC predictors included Grobman’s background variables and two new variables, maternal height and estimated fetal weight. Their overall VBAC success rate was 83.3%. The AUC of Grobman’s model was 0.831 (95% CI: 0.775 to 0.886), and the AUC of their own modified model with two new variables added was 0.857 (sensitivity =72.2%, specificity =83.8%). However, the difference between the AUC of the two models was not significant (Z=-1.69, p=0.091). Hence, they found that Grobman’s model was well accepted in the Chinese population, also that the modified model supplemented with maternal height and estimated fetal weight needed to be further studied in the Chinese population.

There was no case of uterine rupture in the present study, whereas the incidence was 0.90% in the study by Patel et al.^(12)^ and 0.28% in the study by Xing et al.^(15)^ A recent meta-analysis suggested that measurement of lower uterine segment (LUS) thickness antenatally in women with a previous caesarean delivery could be used to predict the occurrence of a uterine defect (scar dehiscence or scar rupture) in women undergoing VBAC^(16)^. Further prospective observational studies are needed using a standard method of intrapartum LUS thickness measurement to predict the outcome of TOLAC and risk of uterine rupture.

The present study included a very important variable i.e. Bishop’s score that has not been incorporated in any of the popular models by Flamm and Geiger^(6)^ and Grobman et al.^(8)^, who included only the individual components of Bishop’s score such as cervical dilation, effacement, and station. Metz et al.^(10)^ and Xing et al.^(15)^ included Bishop’s score in their model, despite it being a subjective variable, its importance was highlighted in their study also^(10,15)^. Metz et al.^ (10)^. used its value as the main factor in developing a score to which a value of 2 to 4 was added for another four variables (history of vaginal delivery, BMI, primary cesarean delivery because of nonrecurring indication, maternal age <35 years) to get the final VBAC score. In the present study, it has an adjusted odds ratio of 3.08 and has a very strong association with successful VBAC (p<0.05).

Spontaneous onset of labour and parity are two other important variables that may be incorporated in the present model and a further study can be planned. There are insufficient studies about VBAC prediction models and most studied only individual variables. Other variables studied in other prediction models are weight gain in pregnancy, preeclampsia, gestational diabetes, insurance. and race^(8,9,15)^. There is a need for the development of a standard prediction model and further studies of this model and many more such models with different permutations and combinations of various variables are required to help predict the success of TOLAC with high accuracy.

### Study Limitations

The small sample size is the limitation of the present study. The study was approved by the institutional ethics committee and was performed in accordance with the ethical standards described in an appropriate version of the 1975 Declaration of Helsinki, as revised in 2000. There is no conflict of interest among the authors and this study was not funded by any organization.

## Conclusion

The present study tests a new VBAC prediction model and shows that it is a good tool for predicting VBAC and hence can be used to counsel women regarding the mode of delivery in current and subsequent pregnancies. Parity, spontaneous onset of labour, admission Bishop’s score, gestational age, and BMI were the factors with a statistically significant association with successful VBAC. Further studies could be proposed such as the comparison of two different types of scoring systems, each system with different variables, in a given population.

## Figures and Tables

**Table 1:**
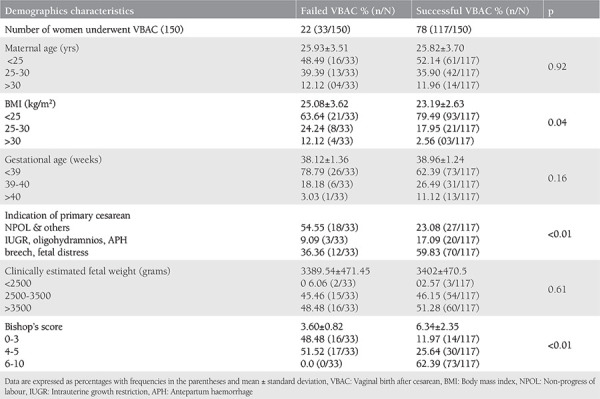
Demographic characteristics of women undergoing trial of labour (*chi-square and Student’s t-test as appropriate)

**Table 2:**
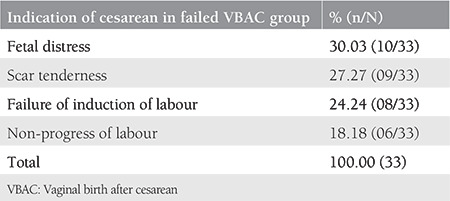
Indication of cesarean section in failed VBAC group

**Table 3:**
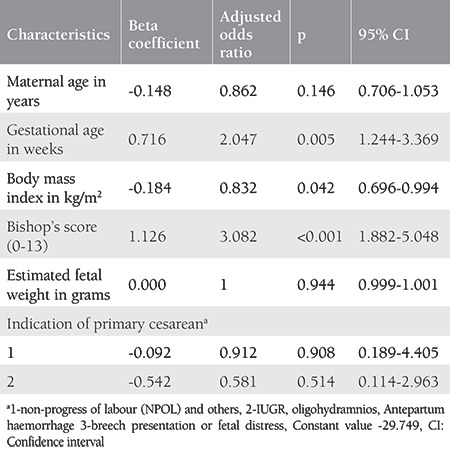
Multivariate regression analysis of the variables included in the prediction model

**Table 4:**
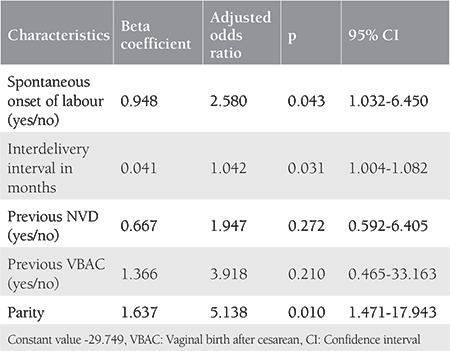
Multivariate regression analysis of additional variables not included in the prediction model
